# Electromagnetic characteristics of *in vivo* nerve fibers at the terahertz-far-infrared band

**DOI:** 10.3389/fbioe.2022.1055232

**Published:** 2022-11-10

**Authors:** Lianghao Guo, Duo Xu, Kaicheng Wang, Yuankun Sun, Qin Zhang, Hui Ning, Chang Lu, Shaomeng Wang, Yubin Gong

**Affiliations:** ^1^ School of Electronic Science and Engineering, University of Electronic Science and Technology of China, Chengdu, Sichuan, China; ^2^ Department of Electronic Communication and Technology, Shenzhen Institute of Information Technology, Shenzhen, China; ^3^ National Key Lab on Vacuum Electronics, Medico-Engineering Cooperation on Applied Medicine Research Center, School of Electronic Science and Engineering, University of Electronic Science and Technology of China, Chengdu, China

**Keywords:** THz-FIR, myelin sheath, nodes of Ranvier, transmission, mode matching algorithm

## Abstract

How terahertz signals perform in the neural system has attracted widespread interest in the life sciences community. Relevant experimental reveals that in animal nerve cells, the myelin sheath of the nerve axon has a higher refractive index than the intracellular and extracellular fluids in the Terahertz-far-infrared (THz-FIR) frequency band. This makes THz-FIR wave transmission possible in nerve fibers. Based on this premise, this article carries out the following work from the theoretical level to investigate the electromagnetic (EM) characteristics of *in vivo* nerve fibers at the THz-FIR band. First, the EM transmission model of the nerve fibers is established and studied theoretically. The dispersion curves of THz-FIR wave modals transmission in nerve fibers are calculated, which predict that nerve fibers can act as dielectric waveguides for transmitting THz-FIR waves and the THz-FIR waves can transmit at speeds up to 10^8^ m/s. Second, a mode matching algorithm is proposed, which is named RNMMA, to calculate the transmission characteristics of THz-FIR waves at the nodes of Ranvier. The scattering matrix obtained from the proposed algorithm is in good agreement with the results from EM simulation software, which reveals how THz-FIR signals are transmitted forward through the nodes of Ranvier with low loss.

## Introduction

As a complex physical system, the human brain has often been a popular topic in life science research, especially in the study of neural information generation and transmission in neurobiology (Adolphs, 2015; Liu, 2018). Electrochemical theory played a leading role in the early studies, although it could not perfectly explain many underlying problems, such as the generation and origin of consciousness (Tononi, 2004; Tegmark, 2015). Meanwhile, action potential signaling mainly relies on the transmembrane transport of ions to cause the potential difference between the excitatory site and the non-excited site, thereby forming a local current. However, traditional electrochemical theories cannot explain the transmission mechanism of high-frequency signals in living organisms, such as how THz and FIR signals that are generated by various redox reactions in biological cells are transmitted. In this context, research of the physical mechanisms that coexist with existing electrochemical theories may reveal new prospects for explaining high-level signal transduction mechanisms, including whether there are more efficient physical ways to transmit and store information in the nervous system. Neuro-electromagnetics provides a new way to study the mechanism of the high-level communication and rapid response of the nervous system. However, it also has many problems, such as how to generate and transmit signals efficiently.

Recently, studies on THz-FIR spectroscopy have confirmed that many oxidative metabolic processes in biological cells produce a large number of biological photons, covering the spectrum range of THz-FIR. Many of the biological macromolecules that play an important role in life activities, such as proteins and phospholipids, also have vibration frequencies in the THz-FIR range (Wetzel and LeVine, 1999; Laman et al., 2008; Miller and Dumas, 2010). The ion channels on the surface of the nerve membrane are not only related to action potential activity but also show that the process of ion transport across the membrane generates THz-FIR radiation (Li et al., 2021; Liu et al., 2021; Guo et al., 2022; Hu et al., 2022; Wang et al., 2022). These are several potential sources for the THz-FIR signals transmitting on the nerve fibers. The THz-FIR signals can also affect the activity of the proteins in the cells (Chao et al., 2021), DNA molecular unhelix (Wu et al., 2020), and the selective transmission of ion channels (Li et al., 2021). As information carrier candidates, THz biophotons may become high-speed signal carriers that connect intracellular organelles and cells independently of electrical and chemical transmissions (Xiang et al., 2020).

Some nerve fibers in the vertebrate nervous system are a kind of EM waveguide structure (i.e., wrapped by high resistance and the low capacitance of nerve myelin sheath). This structure can accelerate the conduction of action potential. Meanwhile, in the FIR range, it supports biological photon signal transmission between cells (Kumar et al., 2016; Zangari et al., 2018; Liu et al., 2019). This natural waveguide structure enables signal transmission at a speed of up to 10^8^ m/s, which is far higher than the conduction speed of the action potential. The nerve myelin sheath with a dense membrane structure can isolate the polar water environment on both sides of the membrane and greatly reduce the attenuation of the THz-FIR wave due to polarization loss in the transmission process.

**FIGURE 1:**
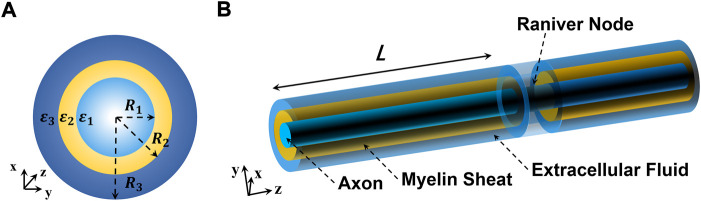
Schematic diagram of the nerve fiber structure. **(A)** Cross section of the nerve fiber and **(B)** 3D perspective graphics of the nerve fiber.

Our current work is focused on how THz-FIR waves behave in nerve fibers, and how the THz-FIR waves hop and exchange energy with periodic nodes of Ranvier structures. In view of this hypothesis, we build a transmission model of nerve fibers and nodes of Ranvier (as shown in Figure 1). The myelinated nerve axon can be regarded as a fiber transmission line that is loaded with layered annular media (i.e., nerve cell fluid, myelin sheath, and extracellular fluid). In contrast to optical fiber, the energies of THz-FIR waves concentrate in the myelin sheath area rather than the fiber core area because of its high dielectric constant; while the physical environment of the nerve fibers are polarity solutions, and there is no obvious boundary. Meanwhile, the node of Ranvier is a periodic structure that is formed by the discontinuity of the myelin sheath, which will inevitably produce energy changes. The main work and innovations of this article are as follows: 1) the field equations and dispersion curves of THz-FIR wave transmission in both myelinated and unmyelinated bare nerve fibers are obtained; 2) the transmission efficiencies of THz-FIR electromagnetic waves in different modes are quantitatively analyzed; and 3) a new mode matching algorithm is proposed that can quickly calculate the scattering parameters and energy exchange of THz-FIR wave transmission at the nodes of Ranvier.

## Methods

### THz-FIR transmission modes in myelin-sheathed nerve fibers

As shown in Figure 1, the nerve fiber is structurally composed of three regions, and the dielectric constants are represented by $\varepsilon_{1}$ , $\varepsilon_{2}$ , and $\varepsilon_{3}$ , respectively. Compared with the intracellular fluid and extracellular fluid of nerve cells (i.e., water as the solvent and have strong absorption characteristics in the THz-FIR band), the myelin sheath is a tight membrane structure with a high dielectric constant and low loss in the THz-FIR band (Liu et al., 2019). The relationship of the dielectric constant of the three-layer media can be expressed as $\varepsilon_{3}<\varepsilon_{1}<\varepsilon_{2}$ (Friede and Bischhausen, 1982; Kumar et al., 2016; Liu et al., 2019).

From the completeness of the EM field, it can be known that the THz-FIR transmission modes on the nerve fiber are composed of the guided modes of the finite mode and the integral of the radiation modes over their continuum spectra. Hence, we decompose the fields on the nerve fiber into two parts,

$$E\left(r,\varphi ,z,t\right)=\overset{N}{\underset{n=1}{\sum }}C_{n}E_{n}\left(r,\varphi \right)e^{j\left({\omega t-\beta }_{z,n}z\right)}+\int C_{\xi }E_{\xi }\left(r,\varphi \right)e^{j\left({\omega t-\beta }_{z,\xi }z\right)}d\xi$$ (1–1)

$$H\left(r,\varphi ,z,t\right)=\overset{N}{\underset{n=1}{\sum }}D_{n}H_{n}\left(r,\varphi \right)e^{j\left({\omega t-\beta }_{z,n}z\right)}+\int D_{\xi }H_{\xi }\left(r,\varphi \right)e^{j\left({\omega t-\beta }_{z,\xi }z\right)}d\xi$$ (1–2)

where the first terms with the subscript *n* represent the guided modes at a certain frequency and the second terms with continuous subscript ξ denote the integral over the radiation modes. Here, the integration variable ξ represents the radial wavenumbers of the radiation modes. *N* is the total number of the guided modes on the nerve fiber at a certain frequency. $C_{n}$ is the normalized amplitude, ω is the angular frequency, and $\beta_{z,n}$ and $\beta_{z,\xi }$ are the propagation constants of the *n*th or ξ th mode along the nerve fiber and have the same value in the three regions. $E_{n}\left(r,\varphi \right)$ , $H_{n}\left(r,\varphi \right)$ describe the electric and magnetic fields of the *n*th mode in the cross-sectional area of the nerve fiber, which can be expressed as the superpositions of transverse and longitudinal components,

$$E_{n}\left(r,\varphi \right)=E_{t,n}\left(r,\varphi \right)+E_{z,n}\left(r,\varphi \right),\;{\;H}_{n}\left(r,\varphi \right)=H_{t,n}\left(r,\varphi \right)+H_{z,n}\left(r,\varphi \right)$$ (2)

The longitudinal components of the electric field $E_{z,n}\left(r,\varphi \right)$ and magnetic field $H_{z,n}\left(r,\varphi \right)$ satisfy the Helmholtz equation. Thus, we have

$$\begin{array}{c} \nabla^{2}E_{z}+\omega^{2}\mu \varepsilon E_{z,n}-j\omega \mu \sigma E_{z,n}=0,\\ \nabla^{2}H_{z}+\omega^{2}\mu \varepsilon H_{z,n}-j\omega \mu \sigma H_{z,n}=0 \end{array}$$ (3)

which can be further expressed as,

$$\begin{array}{c} E_{\text{iz},n}=C_{\text{in}}\psi_{\text{in}}\left(\beta_{\text{in}}r\right)\left(\begin{array}{c} \sin n\varphi \\ \cos n\varphi \end{array}\right),i=1,2,3,\\ H_{\text{iz},n}=D_{\text{in}}\psi_{\text{in}}\left(\beta_{\text{in}}r\right)\left(\begin{array}{c} \sin n\varphi \\ \cos n\varphi \end{array}\right),i=1,2,3 \end{array}$$ (4)

where $\psi_{in}$ represents the Bessel function of order *n* and has different combinations and representations in different regions. The subscript *i* represents *i*th region in Figure 1A. $\beta_{in}$ represents the transverse propagation constant in the *i*th region.

Here, we make the approximation $\varepsilon_{1}\approx \varepsilon_{3}$ , due to the influence of the high dielectric constant myelin sheaths. However, for the bare nerve axon at the node of Ranvier, the approximation is no longer used because the bare axon is similar to a weakly conducting fiber (which we will discuss in detail later on). For the guided modes in the myelinated axons, the propagation constants $\beta_{z}$ satisfy the relations of $k_{0}\sqrt{e_{1}}<\beta_{z}<k_{0}\sqrt{e_{2}}$ . The transverse propagation constants of the three regions can be expressed as $\beta_{1n}^{2}=\beta_{z,n}^{2}-k_{0}^{2}\varepsilon_{1},\beta_{2n}^{2}=k_{0}^{2}\varepsilon_{2}-\beta_{z,n}^{2},$ and $\beta_{3n}^{2}=\beta_{z,n}^{2}-k_{0}^{2}\varepsilon_{3}$ , where $k_{0}=\omega /c_{0}$ . Accordingly, the Bessel function distribution of the corresponding region satisfies $\psi_{1n}=I_{n}\left(\beta_{1n}r\right)$ , $\psi_{2n}=J_{n}\left(\beta_{2n}r\right)+Y_{n}\left(\beta_{2n}r\right)$ , and $\psi_{3n}=K_{n}\left(\beta_{1n}r\right)$ , respectively, which means that the field decays exponentially at both sides of the myelin sheath region. The transverse field components are expressed in terms of the longitudinal field components,

$$E_{t}=\frac{j}{\omega^{2}\mu_{0}\varepsilon_{0}\varepsilon_{i}{-\beta }_{z}^{2}}\left({-\beta }_{z}\nabla_{t}H_{z}+{\omega \mu }_{0}e_{z}\times \nabla_{t}H_{z}\right),\;H_{t}=\frac{j}{\omega^{2}\mu_{0}\varepsilon_{0}\varepsilon_{i}{-\beta }_{z}^{2}}\left({-\beta }_{z}\nabla_{t}E_{z}+{\omega \varepsilon }_{0}\varepsilon_{i}e_{z}\times \nabla_{t}E_{z}\right)$$ (5)

Furthermore, we can obtain the guided mode expressions of 12 field components in the three regions, as follows:

$$\left[\begin{array}{l} E_{1r}\\ E_{1\varphi }\\ H_{1r}\\ H_{1\varphi } \end{array}\right]=j\left[\begin{array}{cc} \begin{array}{l} \frac{\beta_{z,n}}{\beta_{1n}}I_{n}^{\prime }\left(\beta_{1n}r\right)\sin n\varphi \\ \frac{\beta_{z,n}n}{\beta_{1n}^{2}r}I_{n}\left(\beta_{1n}r\right)\cos n\varphi \\ -\frac{{\omega \varepsilon }_{0}\varepsilon_{1}n}{\beta_{1n}^{2}r}I_{n}\left(\beta_{1n}r\right)\cos n\varphi \\ \frac{{\omega \varepsilon }_{0}\varepsilon_{1}}{\beta_{1n}}I_{n}^{\prime }\left(\beta_{1}r\right)\sin n\varphi \end{array} & \begin{array}{l} -\frac{\omega \mu n}{\beta_{1}^{2}r}I_{n}\left(\beta_{1n}r\right)\sin n\varphi \\ -\frac{\omega \mu }{\beta_{1n}}I_{n}^{\prime }\left(\beta_{1n}r\right)\cos n\varphi \\ \frac{\beta_{z,n}}{\beta_{1n}}I_{n}^{\prime }\left(\beta_{1n}r\right)\cos n\varphi \\ -\frac{\beta_{z}n}{\beta_{1n}^{2}r}I_{n}\left(\beta_{1n}r\right)\sin n\varphi \end{array} \end{array}\right]\left[\begin{array}{l} C_{1}\\ D_{1} \end{array}\right]$$ (6a)

$$\left[\begin{array}{l} E_{2r}\\ E_{2\varphi }\\ H_{2r}\\ H_{2\varphi } \end{array}\right]=j\left[\begin{array}{cccc} -\frac{\beta_{z,n}}{\beta_{2n}}J_{n}^{\prime }\left(\beta_{2n}r\right)sinn\varphi & -\frac{\beta_{z,n}}{\beta_{2n}}Y_{n}^{\prime }\left(\beta_{2n}r\right)\sin n\varphi & \frac{\omega \mu n}{\beta_{2n}^{2}r}J_{n}\left(\beta_{2n}r\right)\sin n\varphi & \frac{\omega \mu n}{\beta_{2n}^{2}r}Y_{n}\left(\beta_{2n}r\right)\sin n\varphi \\ -\frac{\beta_{z,n}n}{\beta_{2n}^{2}r}J_{n}\left(\beta_{2n}r\right)cosn\varphi & -\frac{\beta_{z,n}n}{\beta_{2n}^{2}r}Y_{n}\left(\beta_{2n}r\right)\cos n\varphi & \frac{\omega \mu }{\beta_{2n}}J_{n}^{\prime }\left(\beta_{2n}r\right)\cos n\varphi & \frac{\omega \mu }{\beta_{2n}}Y_{n}^{\prime }\left(\beta_{2n}r\right)\cos n\varphi \\ \frac{{\omega \varepsilon }_{0}\varepsilon_{2}n}{\beta_{2n}^{2}r}J_{n}\left(\beta_{2n}r\right)cosn\varphi & \frac{{\omega \varepsilon }_{0}\varepsilon_{2}n}{\beta_{2n}^{2}r}Y_{n}\left(\beta_{2n}r\right)\cos n\varphi & -\frac{\beta_{z,n}}{\beta_{2n}}J_{n}^{\prime }\left(\beta_{2n}r\right)\cos n\varphi & -\frac{\beta_{z,n}}{\beta_{2n}}Y_{n}^{\prime }\left(\beta_{2n}r\right)\cos n\varphi \\ -\frac{{\omega \varepsilon }_{0}\varepsilon_{2}}{\beta_{2n}}J_{n}^{\prime }\left(\beta_{2n}r\right)sinn\varphi & -\frac{{\omega \varepsilon }_{0}\varepsilon_{2}}{\beta_{2n}}Y_{n}^{\prime }\left(\beta_{2n}r\right)\sin n\varphi & \frac{\beta_{z,n}n}{\beta_{2n}^{2}r}J_{n}\left(\beta_{2n}r\right)\sin n\varphi & \frac{\beta_{z,n}n}{\beta_{2n}^{2}r}Y_{n}\left(\beta_{2n}r\right)\sin n\varphi \end{array}\right]\left[\begin{array}{l} C_{21}\\ C_{22}\\ D_{21}\\ D_{22} \end{array}\right]$$ (6b)

$$\left[\begin{array}{l} E_{3r}\\ E_{3\varphi }\\ H_{3r}\\ H_{3\varphi } \end{array}\right]=j\left[\begin{array}{cc} \begin{array}{l} \frac{\beta_{z,n}}{\beta_{3n}}K_{n}^{\prime }\left(\beta_{3n}r\right)\sin n\varphi \\ \frac{\beta_{z,n}n}{\beta_{3n}^{2}r}K_{n}\left(\beta_{3n}r\right)\cos n\varphi \\ -\frac{{\omega \varepsilon }_{0}\varepsilon_{3}n}{\beta_{3n}^{2}r}K_{n}\left(\beta_{3n}r\right)\cos n\varphi \\ \frac{{\omega \varepsilon }_{0}\varepsilon_{3}}{\beta_{3n}}K_{n}^{\prime }\left(\beta_{1}r\right)\sin n\varphi \end{array} & \begin{array}{l} -\frac{\omega \mu n}{\beta_{3n}^{2}r}K_{n}\left(\beta_{3n}r\right)\sin n\varphi \\ -\frac{\omega \mu }{\beta_{3n}}K_{n}^{\prime }\left(\beta_{3n}r\right)\cos n\varphi \\ \frac{\beta_{z,n}}{\beta_{3n}}K_{n}^{\prime }\left(\beta_{3n}r\right)\cos n\varphi \\ -\frac{\beta_{z,n}n}{\beta_{3n}^{2}r}K_{n}\left(\beta_{3n}r\right)\sin n\varphi \end{array} \end{array}\right]\left[\begin{array}{l} C_{3}\\ D_{3} \end{array}\right]$$ (6c)

The dispersion curves of THz-FIR wave transmitting in nerve fibers can be obtained by using the tangential continuous boundary conditions for the electric and magnetic fields on $r=R_{1}$ and $r=R_{2}$ , and the dispersion equation of *n*th HEM mixing mode is as follows:

$$\left|\begin{array}{cccccccc} I_{n}\left(\beta_{1n}R_{1}\right) & 0 & {-J}_{n}\left(\beta_{2n}R_{1}\right) & {-Y}_{n}\left(\beta_{2n}R_{1}\right) & 0 & 0 & 0 & 0\\ 0 & I_{n}\left(\beta_{1n}R_{1}\right) & 0 & 0 & {-J}_{n}\left(\beta_{2n}R_{1}\right) & {-Y}_{n}\left(\beta_{2n}R_{1}\right) & 0 & 0\\ \frac{\beta_{z}n}{\beta_{1n}^{2}R_{1}}I_{n}\left(\beta_{1n}R_{1}\right) & -\frac{\omega \mu }{\beta_{1n}}I_{n}^{\prime }\left(\beta_{1n}R_{1}\right) & \frac{\beta_{z}n}{\beta_{2n}^{2}R_{1}}J_{n}\left(\beta_{1n}R_{1}\right) & \frac{\beta_{z}n}{\beta_{2n}^{2}R_{1}}Y_{n}\left(\beta_{2n}R_{1}\right) & -\frac{\omega \mu }{\beta_{2n}}J_{n}^{\prime }\left(\beta_{2n}R_{1}\right) & -\frac{\omega \mu }{\beta_{2n}}Y_{n}^{\prime }\left(\beta_{2n}R_{1}\right) & 0 & 0\\ \frac{{\omega \varepsilon }_{0}\varepsilon_{1}}{\beta_{1n}}I_{n}^{\prime }\left(\beta_{1}R_{1}\right) & -\frac{\beta_{z}n}{\beta_{1n}^{2}R_{1}}I_{n}\left(\beta_{1n}R_{1}\right) & \frac{{\omega \varepsilon }_{0}\varepsilon_{2}}{\beta_{2n}}J_{n}^{\prime }\left(\beta_{2n}R_{1}\right) & \frac{{\omega \varepsilon }_{0}\varepsilon_{2}}{\beta_{2n}}Y_{n}^{\prime }\left(\beta_{2n}R_{1}\right) & -\frac{\beta_{z}n}{\beta_{2n}^{2}R_{1}}J_{n}\left(\beta_{2n}R_{1}\right) & -\frac{\beta_{z}n}{\beta_{2n}^{2}R_{1}}Y_{n}\left(\beta_{2n}R_{1}\right) & 0 & 0\\ 0 & 0 & J_{n}\left(\beta_{2n}R_{2}\right) & Y_{n}\left(\beta_{2n}R_{2}\right) & 0 & 0 & {-K}_{n}\left(\beta_{3n}R_{2}\right) & 0\\ 0 & 0 & 0 & 0 & J_{n}\left(\beta_{2n}R_{2}\right) & Y_{n}\left(\beta_{2n}R_{2}\right) & 0 & {-K}_{n}\left(\beta_{3n}R_{2}\right)\\ 0 & 0 & -\frac{\beta_{z}n}{\beta_{2n}^{2}R_{2}}J_{n}\left(\beta_{2n}R_{2}\right) & -\frac{\beta_{z}n}{\beta_{2n}^{2}R_{2}}Y_{n}\left(\beta_{2n}R_{2}\right) & \frac{\omega \mu }{\beta_{2n}}J_{n}^{\prime }\left(\beta_{2n}R_{2}\right) & \frac{\omega \mu }{\beta_{2n}}Y_{n}^{\prime }\left(\beta_{2n}R_{2}\right) & -\frac{\beta_{z}n}{\beta_{3n}^{2}R_{2}}K_{n}\left(\beta_{3n}R_{2}\right) & \frac{\omega \mu }{\beta_{3n}}K_{n}^{\prime }\left(\beta_{3n}R_{2}\right)\\ 0 & 0 & -\frac{{\omega \varepsilon }_{0}\varepsilon_{2}}{\beta_{2n}}J_{n}^{\prime }\left(\beta_{2n}R_{2}\right) & -\frac{{\omega \varepsilon }_{0}\varepsilon_{2}}{\beta_{2n}}Y_{n}^{\prime }\left(\beta_{2n}R_{2}\right) & \frac{\beta_{z}n}{\beta_{2n}^{2}R_{2}}J_{n}\left(\beta_{2n}R_{2}\right) & \frac{\beta_{z}n}{\beta_{2n}^{2}R_{2}}Y_{n}\left(\beta_{2n}R_{2}\right) & -\frac{{\omega \varepsilon }_{0}\varepsilon_{3}}{\beta_{3n}}K_{n}^{\prime }\left(\beta_{1}R_{2}\right) & \frac{\beta_{z}n}{\beta_{3n}^{2}R_{2}}K_{n}\left(\beta_{3n}R_{2}\right) \end{array}\right|=0$$ (7)

It is worth noting that when *n*≠0, the aforementioned equation has non-zero solutions, indicating that the THz-FIR waves on the nerve fibers are transmitting in mixed modes. When n=0, the THz-FIR waves will no longer transmit in the mixed modes but are decomposed into independent TE and TM modes, and the field components satisfy $E_{z}=E_{\varphi }=H_{r}=0$ or $H_{z}=H_{\varphi }=E_{r}=0$ . Therefore, in the case of n = 0, the left-hand determinant can be rewritten as a 4 × 4 order determinant.

For the radiation modes, it is used to describe the radiation fields outside and inside of the myelin sheath, which are necessary supplements to the guided modes to provide a completely orthogonal set of modes. The fields of the radiation modes will not decay exponentially for increasing values of *r* outside of the nerve fiber (i.e., extend to infinity). Because there is no need to limit the function describing the radiation modes to those decaying exponentially for large *r*, a combination of Bessel and Neumann functions is used to describe the radiation modes. It is worth noting that the radiation modes also satisfy the boundary conditions and the fields remain finite on-axis at *r* = 0. The propagation constants of radiation modes satisfy $\beta_{z}<k_{0}\sqrt{e_{1}}$ , and the transverse propagation constants can be expressed as $\beta_{1n}^{2}=k_{0}^{2}\varepsilon_{1}-\beta_{z,n}^{2},\beta_{2n}^{2}=k_{0}^{2}\varepsilon_{2}-\beta_{z,n}^{2}$ , and $\beta_{3n}^{2}=k_{0}^{2}\varepsilon_{3}-\beta_{z,n}^{2}$ in three regions. Accordingly, the Bessel function $\psi_{in}$ in Eq. 4 can be written as $\psi_{1n}=J_{n}\left(\beta_{1n}r\right)$ , $\psi_{2n}=J_{n}\left(\beta_{2n}r\right)+Y_{n}\left(\beta_{2n}r\right)$ , and $\psi_{3n}=J_{n}\left(\beta_{3n}r\right)+Y_{n}\left(\beta_{3n}r\right)$ . Unfortunately, in the general field solution of the radiation mode, there are 10 unknowns and only eight boundary conditions. Therefore, the propagation constants of the radiation modes can theoretically take continuous values (Dietrich and Marcuse, 1970; Marcuse, 1973).

Here, we ignore the evanescent radiation modes of the nerve fiber, which are the modes with imaginary values of the propagation constants $\beta_{z}$ and do not propagate in the z-direction. However, omitting evanescent radiation modes keeps the set of orthogonal modes from being complete.

### Nodes of Ranvier mode matching algorithm

**FIGURE 2:**
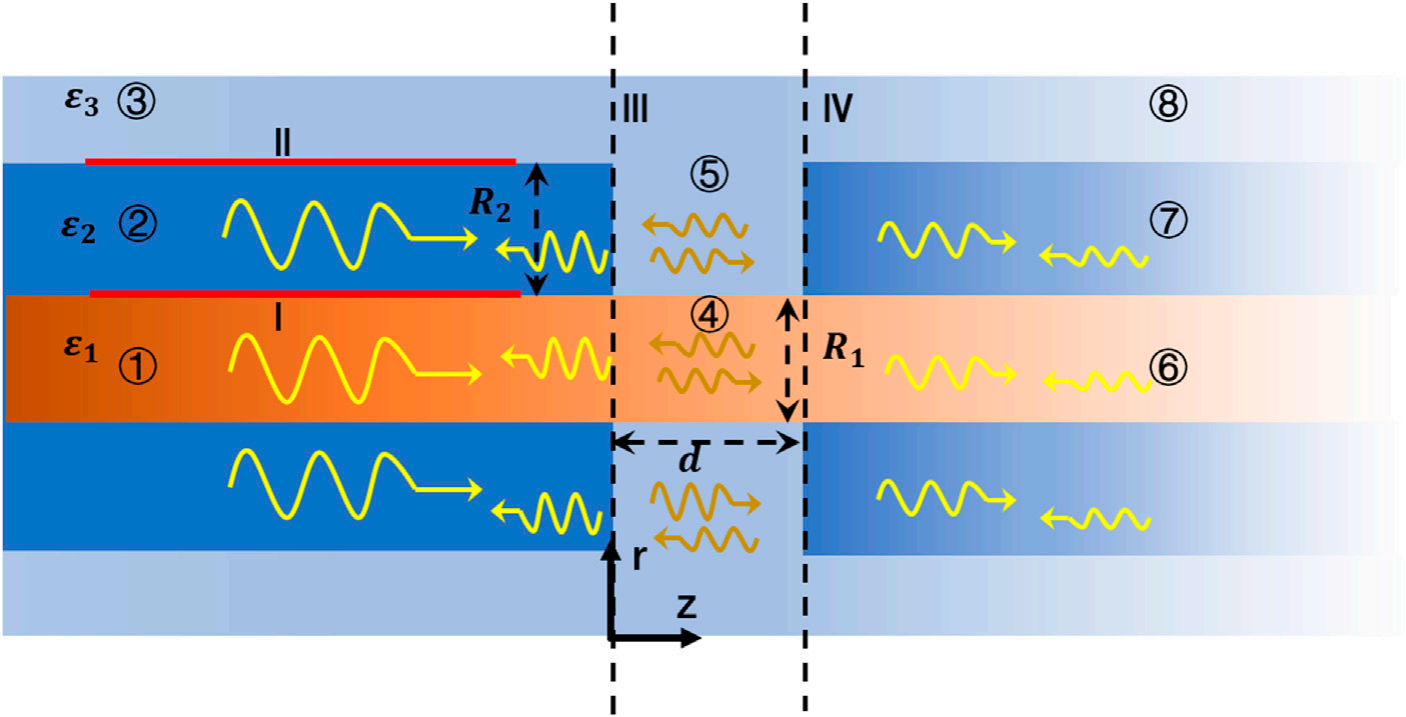
Schematic diagram of one node of Ranvier structure, and electromagnetic wave transmissions and reflections.

In the vertebrate nervous system, nerve fibers are not completely enclosed by myelin sheaths, which periodically disintegrate to form structures called the node of Ranvier. The membranes of the axon that are exposed at the narrow node of Ranvier contain large numbers of ion channels, which enable the action potential to jump over the nodes. A schematic diagram of the structure is shown in Figure 2. The calculations of the electric and magnetic fields at the node of Ranvier are very complicated because of the discontinuity of the longitudinal section of the THz-FIR wave in the transmission direction. To analyze the THz-FIR wave transmission and field mode transformation at the nodes of Ranvier, we propose the nodes of Ranvier pattern matching algorithm (RNMMA), which theoretically consists of three steps: 1) the RNMMA begins by expanding the tangential components of the electric and magnetic fields at the node in terms of the normal modes in each region; 2) the scattering matrices on the boundaries of III and IV are obtained by using mode orthogonality and power normalization; and 3) the total scattering matrix of one node of Ranvier can be obtained from the matrix cascade, and then the distributions and mode characteristics of the electric and magnetic fields at the nodes of Ranvier can be solved. To simplify the analysis, we made the boundaries of III and IV perpendicular to the z-direction, while the boundaries are not strictly perpendicular physiologically.

As shown in Figure 2, the structure of the node of Ranvier (④ and ⑤ regions) can be regarded as an unmyelinated neural axon, whose structure is similar to an optical fiber. The permittivity of the extracellular fluid in ⑤ region is the same as that in ③ region, and the permittivity in ④ region is the same as that in ① region. The field components can also be described by Eqs 1–5. The Bessel function in ④ and ⑤ regions can be expressed as $\psi_{4n}=J_{n}\left(\beta_{4n}r\right)$ and $\psi_{5n}=K_{n}\left(\beta_{5n}r\right)$ for guided modes, which can ensure that the field propagates in region ④ and decays exponentially in the r-direction in region ⑤. The propagation constants satisfy $k_{0}\sqrt{\varepsilon_{5}}<\beta_{z,n}<k_{0}\sqrt{\varepsilon_{3}}$ , and the transverse propagation constants can be expressed $\beta_{4n}^{2}=k_{0}^{2}\varepsilon_{4}-\beta_{z,n}^{2}$ and $\beta_{5n}^{2}=\beta_{z,n}^{2}-k_{0}^{2}\varepsilon_{5}$ . When $\beta_{z,n}<k_{0}\sqrt{\varepsilon_{5}}$ , the transmission modes of the THz-FIR wave generated by the node of Ranvier are radiation modes. The transverse propagation constants can be expressed as $\beta_{\;n}^{2}=k_{0}^{2}\varepsilon_{4}-\beta_{z,n}^{2}$ and $\beta_{5n}^{2}=k_{0}^{2}\varepsilon_{5}-\beta_{z,n}^{2}$ . The field components in the r-direction can be described by a combination of Bessel and Neumann functions, $\psi_{4n}=J_{n}\left(\beta_{4n}r\right)$ and $\psi_{5n}=J_{n}\left(\beta_{5n}r\right)+Y_{n}\left(\beta_{5n}r\right)$ .

The tangential components of electric and magnetic fields at the boundary can be expressed in terms of each normal mode:

$$E_{t}=\underset{N,p}{\sum }C_{N}^{p}E_{t,N}^{p}\left(r,\varphi \right)e^{{-\mathrm{jp}\beta }_{\text{zN}}z}$$ (8)

$$H_{t}=\underset{N,p}{\sum }C_{N}^{p}H_{t,N}^{p}\left(r,\varphi \right)e^{{-\mathrm{jp}\beta }_{\text{zN}}z}$$ (9)

where *p* (*p* = +, −) represents the propagation direction of the wave, *N* indicates the mode number, $A_{N}^{p}$ is the amplitude coefficient of each mode, and $E_{t,N}^{p}\left(r,\varphi \right)$ and $H_{t,N}^{p}\left(r,\varphi \right)$ are the transverse electric field and magnetic field of each mode in different regions. The pattern functions of different regions all satisfy the orthogonality theorem of patterns,

$$\oint_{S}e_{z}\cdot \left(E_{t,m}^{q}\times H_{t,n}^{p^{*}}+E_{t,n}^{p^{*}}\times H_{t,m}^{q}\right)\text{ds}={4\delta }_{\mathrm{mn}}S_{0}\left(p,q\right)P_{n}$$ (10)

where $\delta_{mn}$ is the Kronecker delta. When m=n , $\delta_{mn}=1$ , otherwise $\delta_{mn}=0$ , which means the different modes are orthogonal to each other. Meanwhile, p and q represent the propagation directions of the modes. When p and q are both positive, S=1 ; when p and q are both negative, S=−1 ; otherwise, S=0 . This means the same modes of forwarding and backward transmissions are also orthogonal to each other. The scattering matrix (SM) is the premise of analyzing the THz-FIR wave propagation mode at the node of Ranvier, the core of which is establishing the coupling matrix (CM). N and M modes are, respectively, considered in the axon and node of Ranvier regions on both sides of the III boundary. By applying the continuity conditions ( $E_{t,axon}=E_{t,NR}$ and $H_{t,axon}=H_{t,NR}$ ) at the junction (z = 0), we obtain the equation for the electric field as

$$\begin{array}{cc} 0\leq r<R_{1} & \overset{N}{\underset{n=1}{\sum }}\left(C_{1,n}^{+}\pm C_{1,n}^{-}\right)\psi_{1t,n}\left(r,\varphi \right)\\ R_{1}\leq r<R_{2} & \overset{N}{\underset{n=1}{\sum }}\left(C_{2,n}^{+}\pm C_{2,n}^{-}\right)\psi_{2t,n}\left(r,\varphi \right)\\ R_{2}\leq r & \overset{N}{\underset{n=1}{\sum }}\left(C_{3,n}^{+}\pm C_{3,n}^{-}\right)\psi_{3t,n}\left(r,\varphi \right) \end{array}\}=\left\{ \begin{array}{cc} \overset{M}{\underset{m=1}{\sum }}\left(D_{4,m}^{+}\pm D_{4,m}^{+}\right)\psi_{t,m}\left(r,\varphi \right) & 0\leq r<R_{1}\\ \overset{M}{\underset{m=1}{\sum }}\left(D_{5,m}^{+}\pm D_{5,m}^{+}\right)\psi_{5t,m}\left(r,\varphi \right) & R_{1}\leq r \end{array}\right.$$ (11)

where $\psi_{it,n}\left(r,\varphi \right)$ represents tangential electric field (E) and tangential magnetic field (H). By making use of the property of mode orthogonality, we can derive a set of an equation involving the unknown coefficients. To this end, the aforementioned equation is multiplied $\psi_{it,n}\left(r,\varphi \right)$ in the different regions and integrated with the whole interface. We can get

$$\underset{i=1,2,3}{\sum }\overset{N}{\underset{n=1}{\sum }}F_{i,n}\left(C_{i,n}^{+}{+C}_{i,n}^{-}\right)=\underset{j=1,2,3}{\sum }\underset{i=4,5}{\sum }\overset{M}{\underset{m=1}{\sum }}f_{\mathrm{ij},\mathrm{nm}}\left(D_{i,m}^{+}{+D}_{i,m}^{+}\right)$$ (12)

$$\underset{i=1,2,3}{\sum }\overset{N}{\underset{n=1}{\sum }}F_{i,n}^{h}\left(C_{i,n}^{+}{-C}_{i,n}^{-}\right)=\underset{j=1,2,3}{\sum }\underset{i=4,5}{\sum }\overset{M}{\underset{m=1}{\sum }}f_{ij,nm}^{h}\left(D_{i,m}^{+}{-D}_{i,m}^{+}\right)$$ (13)

where $F_{i,n}$ and $f_{ij,nm}$ are the CM, and their representations are as follows:

$$F_{i,n}=\int_{0}^{2\pi }\int_{R_{i-1}}^{R_{i}}\psi_{\mathrm{it},n}^{e}\psi_{\mathrm{it},n}^{e}r\mathrm{drd}\theta ,i=1,2,3$$ (14–1)

$$f_{\text{ji},\text{nm}}=\int_{0}^{2\pi }\int_{R_{j-1}}^{R_{j}}\psi_{\mathrm{jt},m}^{e}\psi_{\mathrm{it},n}^{e}r\mathrm{drd}\theta ,j=4,5\;and\;i=1,2,3$$ (14–2)

$$F_{i,n}^{h}=\int_{0}^{2\pi }\int_{R_{i-1}}^{R_{i}}\psi_{\mathrm{it},n}^{h}\psi_{\mathrm{it},n}^{h}r\mathrm{drd}\theta ,i=1,2,3$$ (14–3)

$$f_{\text{ji},\text{nm}}^{h}=\int_{0}^{2\pi }\int_{R_{j-1}}^{R_{j}}\psi_{\mathrm{jt},n}^{h}\psi_{\mathrm{it},m}^{h}r\mathrm{drd}\theta ,\;j=4,5\;and\;i=1,2,3$$ (14–4)

where $\psi_{it,n}^{e}$ represents the transverse electric field component of the *n*th mode in the *i*th region of the axon region. $\psi_{it,n}^{h}$ represents the transverse magnetic field component of the *n*th mode in the *i*th region of the axon region. Meanwhile, $\psi_{jt,m}^{e}$ represents the transverse electric field component of the *m*th mode in the *j*th region of the NR region. Finally, $\psi_{jt,m}^{h}$ represents the transverse magnetic field component of the *m*th mode in the *j*th region of the NR region.

Therefore, the SM at the Node of Ranvier can be obtained by using the method in Ref. (Itoh, 1989), as follows:

$$S_{22\_\text{III}}={\left(I+M_{y\_\text{III}}^{t}M_{x\_\text{III}}\right)}^{-1}\left(I-M_{y\_\text{III}}^{t}M_{x\_\text{III}}\right)$$ (15-1)

$$S_{21\_\text{III}}=2{\left(I+M_{y\_\text{III}}^{t}M_{x\_\text{III}}\right)}^{-1}M_{y\_\text{III}}^{t}M_{x\_\text{III}}$$ (15-2)

$$S_{12\_\text{III}}=M_{x\_\text{III}}\left(I+S_{22\_\text{III}}\right)$$ (15-3)

$$S_{11\_\text{III}}=M_{x\_\text{III}}S_{21\_\text{III}}-I$$ (15-4)

where

$$M_{x\_III}={\left(Z_{axon}^{2}F_{\mathrm{axon}}\right)}^{-1}\left(f_{NR}Z_{\mathrm{axon}}Z_{\mathrm{NR}}\right)$$ (16-1)

$$M_{y\_III}={\left(Z_{\mathrm{axon}}^{2}F_{\mathrm{axon}}^{h}\right)}^{-1}\left(f_{\mathrm{NR}}^{h}Z_{\mathrm{axon}}Z_{\mathrm{NR}}\right)$$ (16-2)

and I is the unit matrix. Each term in the CM is given by Eq. 14, and its matrix expression is as follows:

$$F_{\text{axon}}={\left[\begin{array}{ccc} F_{1,1}{+F}_{2,1}{+F}_{3,1} & \cdots & 0\\ \vdots & \ddots & \vdots \\ 0 & \cdots & F_{1,N}{+F}_{2,N}{+F}_{3,N} \end{array}\right]}_{\left(N\times N\right)}$$ (17-1)

$$F_{\text{axon}}^{h}={\left[\begin{array}{ccc} {F\;}_{1,1}^{h}{+F\;}_{2,1}^{h}{+F\;}_{3,1}^{h} & \cdots & 0\\ \vdots & \ddots & \vdots \\ 0 & \cdots & {F\;}_{1,N}^{h}{+F\;}_{2,N}^{h}{+F\;}_{3,N}^{h} \end{array}\right]}_{\left(N\times N\right)}$$ (17-2)

$$f_{\text{NR}}={\left[\begin{array}{ccc} f_{41,11}{+f}_{52,11}{+f}_{53,11} & \cdots & f_{41,1M}{+f}_{52,1M}{+f}_{53,1M}\\ \vdots & \ddots & \vdots \\ f_{41,N1}{+f}_{52,N1}{+f}_{53,N1} & \cdots & f_{41,\mathrm{NM}}{+f}_{52,\mathrm{NM}}{+f}_{53,\mathrm{NM}} \end{array}\right]}_{\left(N\times M\right)}$$ (17-3)

$$f_{\text{NR}}^{h}={\left[\begin{array}{ccc} f_{41,11}^{h}{+f}_{52,11}^{h}{+f}_{53,11}^{h} & \cdots & f_{41,1M}^{h}{+f}_{52,1M}^{h}{+f}_{53,1M}^{h}\\ \vdots & \ddots & \vdots \\ f_{41,N1}^{h}{+f}_{52,N1}^{h}{+f}_{53,N1}^{h} & \cdots & f_{41,\mathrm{NM}}^{h}{+f}_{52,\mathrm{NM}}^{h}{+f}_{53,\mathrm{NM}}^{h} \end{array}\right]}_{\left(N\times M\right)}$$ (17-4)

$$Z_{\text{axon}}=\left[\begin{array}{ccc} P_{1,\mathrm{axon}}^{-0.5} & \cdots & 0\\ \vdots & \ddots & \vdots \\ 0 & \cdots & P_{N,\mathrm{axon}}^{-0.5} \end{array}\right]$$ (17-5)

$$Z_{\text{NR}}=\left[\begin{array}{ccc} P_{1,\mathrm{NR}}^{-0.5} & \cdots & 0\\ \vdots & \ddots & \vdots \\ 0 & \cdots & P_{M,\mathrm{NR}}^{-0.5} \end{array}\right]$$ (17-6)

where $Z_{axon}$ and $Z_{NR}$ are the normalized matrices, which can be determined by Eq. 10. The SM of the boundary IV can be solved by the same methods. Then, the SM of the entire NR can be obtained by the cascade formula of two-port networks (Chu and Itoh, 1986).

## Results and discussion

### Dispersion curves and field distributions of different modes

**FIGURE 3:**
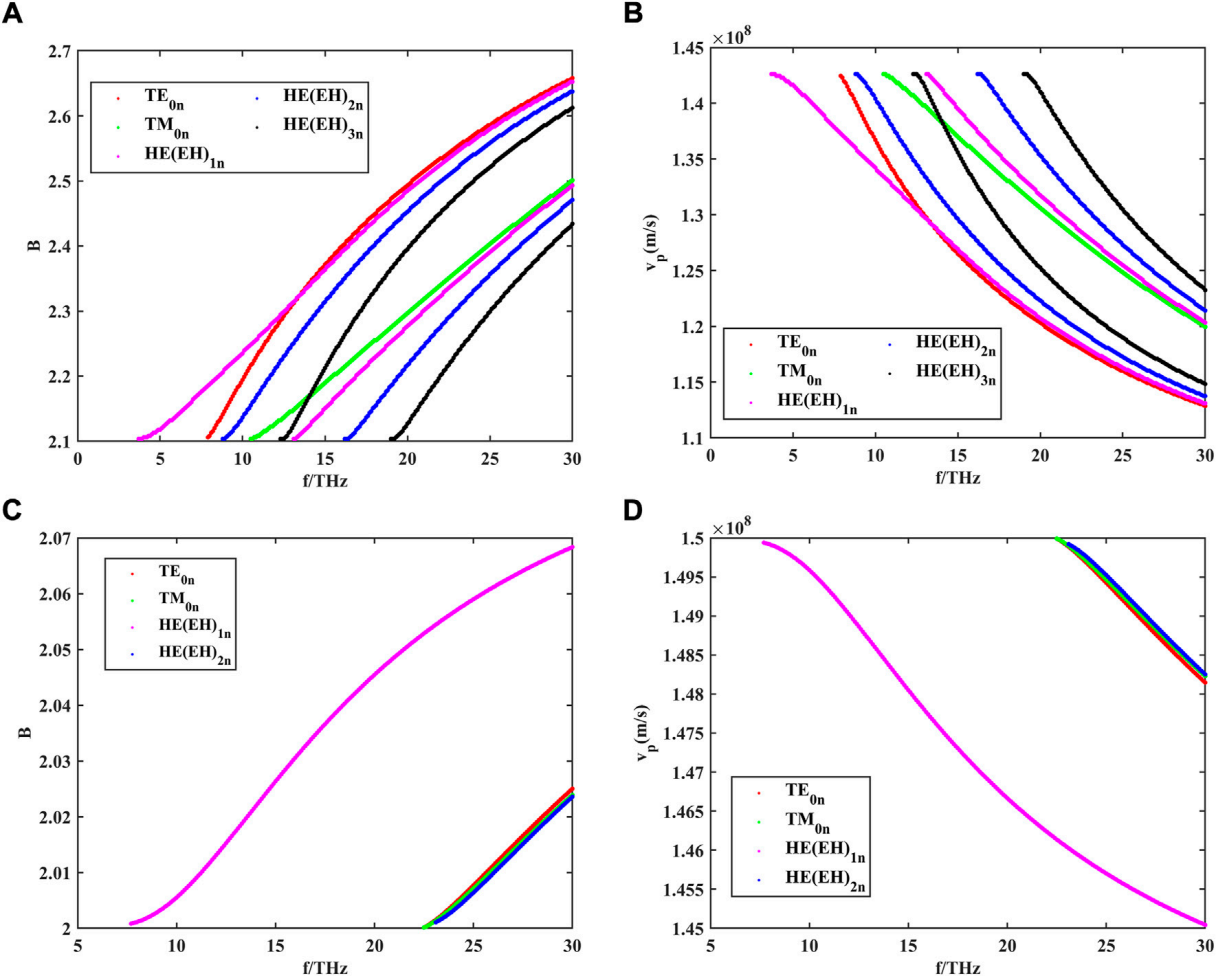
**(A)** Dispersion curves of different guided modes in the myelinated nerve fibers within the frequency range of 0–30 THz, where *m* is the number of angular variations; **(B)** phase velocity of different guided modes in the myelinated nerve fibers within the frequency range of 0–30 THz; **(C)** dispersion curves of different guided modes in the unmyelinated nerve fibers within the frequency range of 0–30 THz; and **(D)** phase velocity of different guided modes in the unmyelinated nerve fibers within the frequency range of 0–30 THz.

The dispersion curves of the guided modes in the nerve fiber can be obtained by solving Eq. 7. The results are shown in Figure 3. Here, the radii of the three regions of the model are set at $R_{1}=8\mu m$ , $R_{2}=10\mu m$ , and $R_{3}=20\mu m$ (Kwon et al., 2017). The refractive indexes of the medium in the three regions are set as $n_{1}=2.1$ , $n_{2}=3$ , and $n_{3}=2$ (Liu et al., 2019), and the dielectric constants can be expressed as $\varepsilon_{i}=n_{i}^{2}$ . Meanwhile, we will further investigate the effect of the difference in the dielectric constant between the myelin sheath, and the internal and external fluids on the transmission of the THz-FIR wave.

*B* is the relative propagation constant, which can be represented as $B=\beta_{z}/k_{0}$ . $k_{0}$ is the vacuum propagation constant and is expressed as $k_{=0}=\omega \sqrt{\varepsilon_{0}\mu_{0}}$ . As shown in Figure 3, we only show the case of *m* = 0, 1, 2, and 3, but in practice the mode order *n* can take any value. Because the outer boundary of the nerve fiber is a metal tube wall with incomplete conductance, the tangential electric field on the outer boundary surface is not zero and the transmitted electromagnetic waves will no longer be pure TE mode or TM mode. Similar to the structure of optical fibers, the main transmission mode of terahertz waves in myelinated nerve fibers is mainly HEM mixed mode. According to the dominance of electric and magnetic fields, the modes obtained under the nerve fiber are denoted as the HEmn and EHmn hybrid modes when m > 1 (or TE0n or TM0n modes).

As can be seen from the dispersion curves in Figures 3A, C, both myelinated and unmyelinated nerve fibers transmit independent patterns, and each mode has a different propagation constant and corresponding transverse cutoff wavenumber at a given frequency. Hence, for any given excitation signal, the mode that satisfies the transmission characteristics can be found by the dispersion curve. Meanwhile, axons encased in high-refractive myelin sheaths greatly increase the number of modes that can be transmitted in nerve fibers compared to bare axons. By using Eq. 18, we further investigated the phase velocities of different modes of propagation in myelin-wrapped and bare axons. The calculated results are shown in Figures 3B, D. The transmission speed of THz waves in nerve fibers can reach 108 m/s, which is much higher than the propagation speed of action potential in myeloid axons (order of magnitude 102 m/s). Therefore, this unique biological structure is more conducive to supporting long-distance and high-speed communication between nerve cells.

$$v_{p}=\frac{\omega }{\beta_{z}}$$ (18)

**FIGURE 4:**
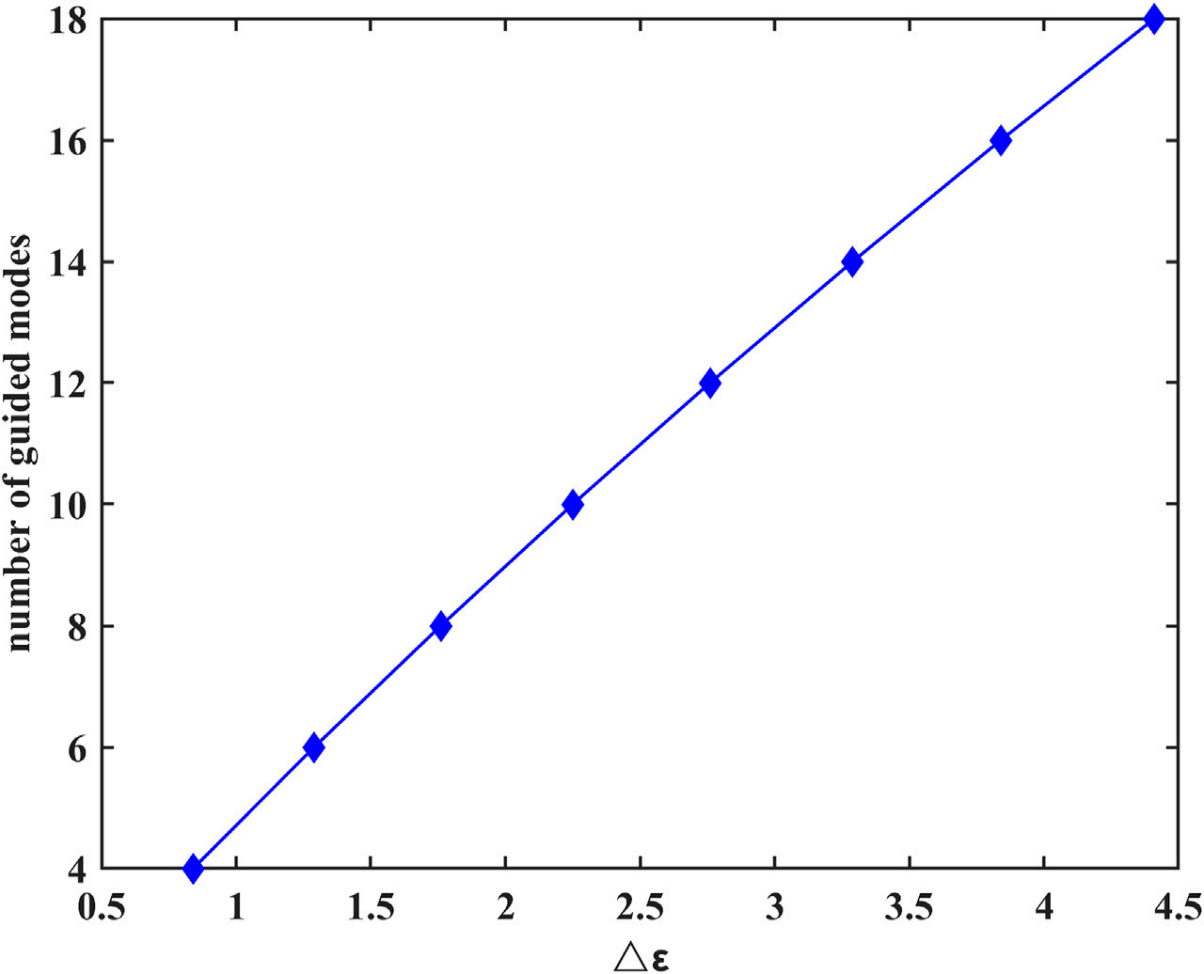
Difference between the dielectric constant of the myelin sheath and internal fluid on the number of guided modes at f = 30 THz.

To further analyze the number of guided modes that nerve fibers can transmit, we investigate the effect of the difference between the dielectric constant of the myelin sheath and internal fluid on the number of guided modes when f = 30 THz. The results are shown in Figure 4.

**FIGURE 5:**
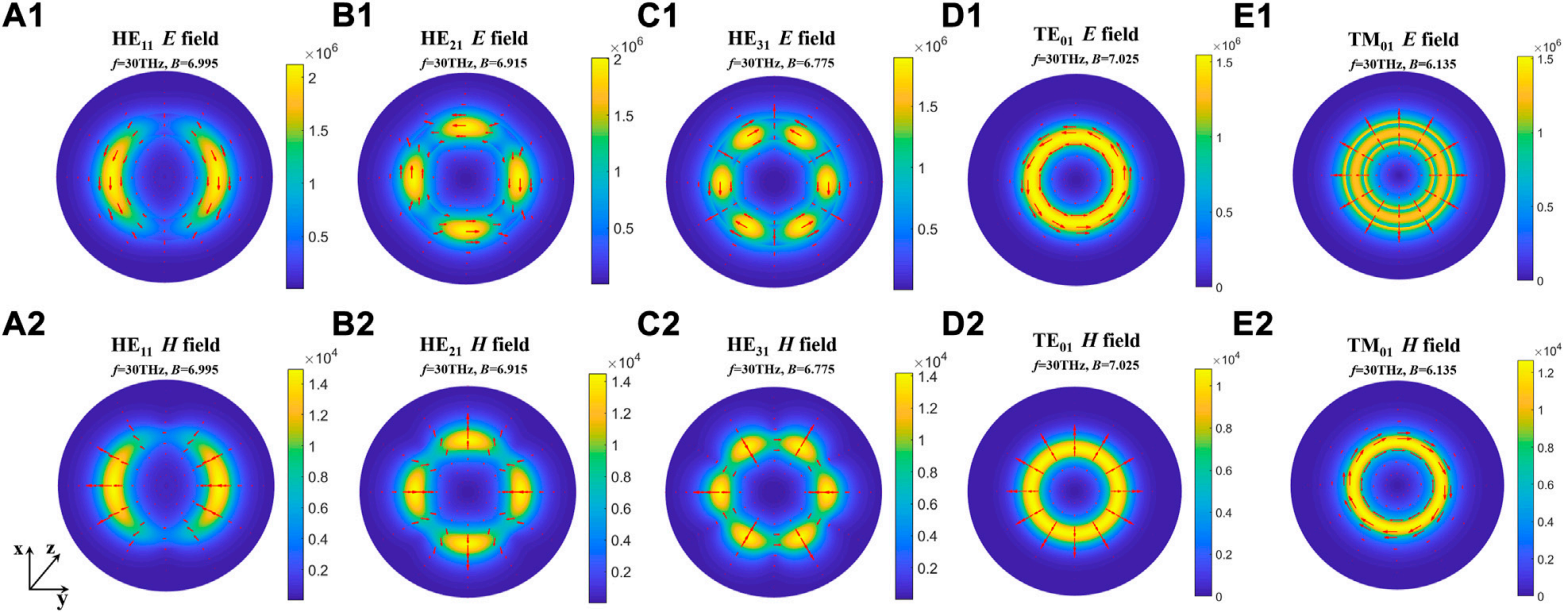
The lateral field distributions of different modes. **(A1-E1)** Electric field intensities and their vector diagrams of the first five modes; **(A2-E2)** magnetic field intensities and their vector diagrams of the first five modes.

The results show that the number of guided modes increases with the increase of the difference between the dielectric constants of the myelin sheath and internal fluid, which indicates that the nerve fiber can transmit THz signals to a greater extent in the frequency band with a higher permittivity of the myelin sheath. In addition, the lateral field distributions of different modes can be obtained by taking the results of Figure 3 into Eq. 6, the results are shown in Figure 5. Here, Figures 5A1–E1 show the electric field intensities and their vector diagrams of the first five modes of THz-FIR wave transmission at the nerve fiber port. Figures 5A2–E2 show the magnetic field intensities and their vector diagrams.

**FIGURE 6:**
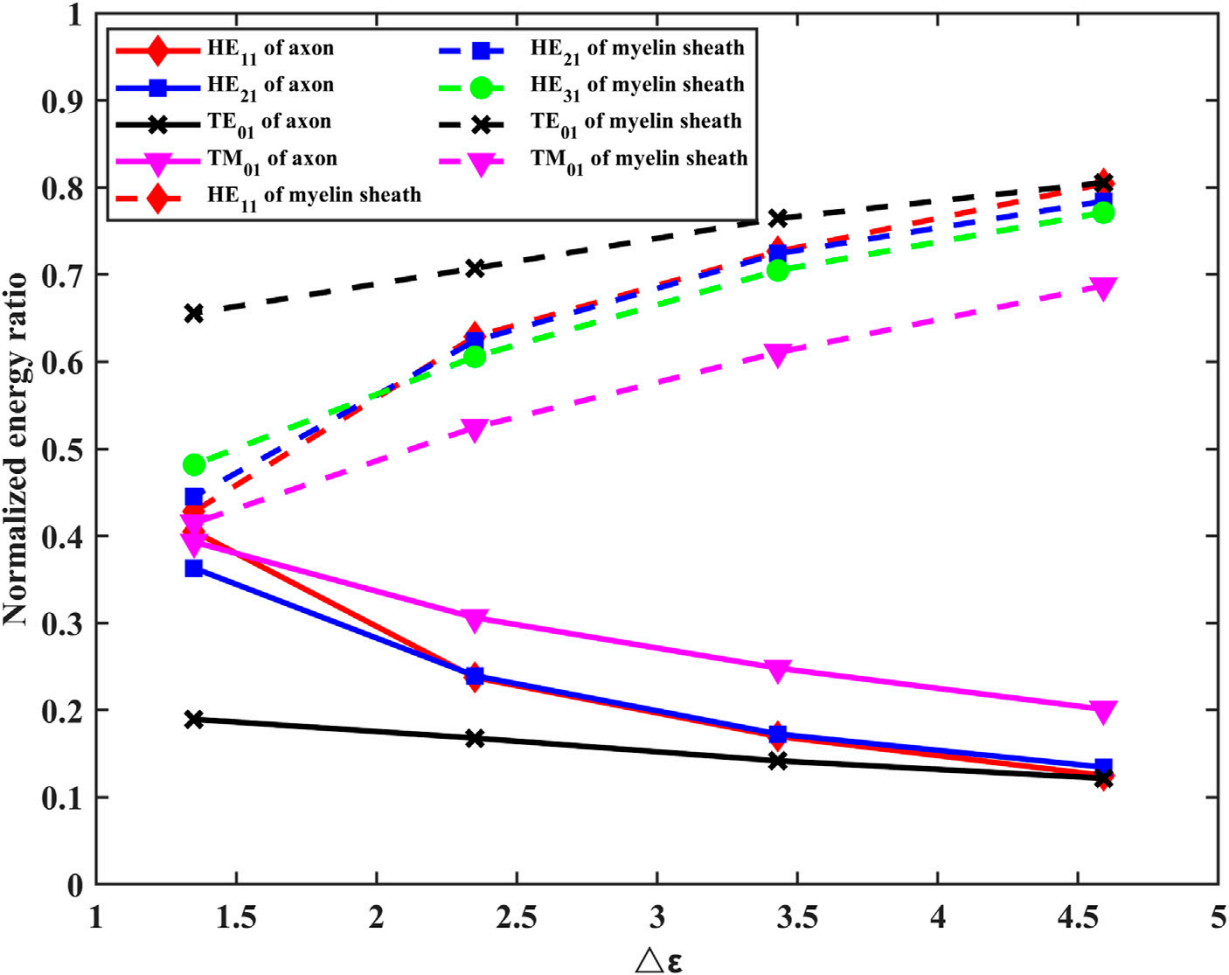
Effects of changes in the permittivity of myelin sheaths on energy distribution in axons, myelin sheaths, and fluid regions.

The results show that the energies of electric and magnetic fields are mainly concentrated in the myelin region in both fundamental and higher modes. To compare the influence of different myelin dielectric constants and modes on the energy distribution, we conducted the following supplementary studies. The results are shown in Figure 6.

It can be seen from Figure 6 that the energy in the myelinated region increases as the difference in dielectric constants increases. At the same frequency, lower-order modes with high propagation constants concentrate energy more efficiently in the myelin sheath region.

## Transmission characteristics of THz-FIR waves in nerve fibers

**FIGURE 7:**
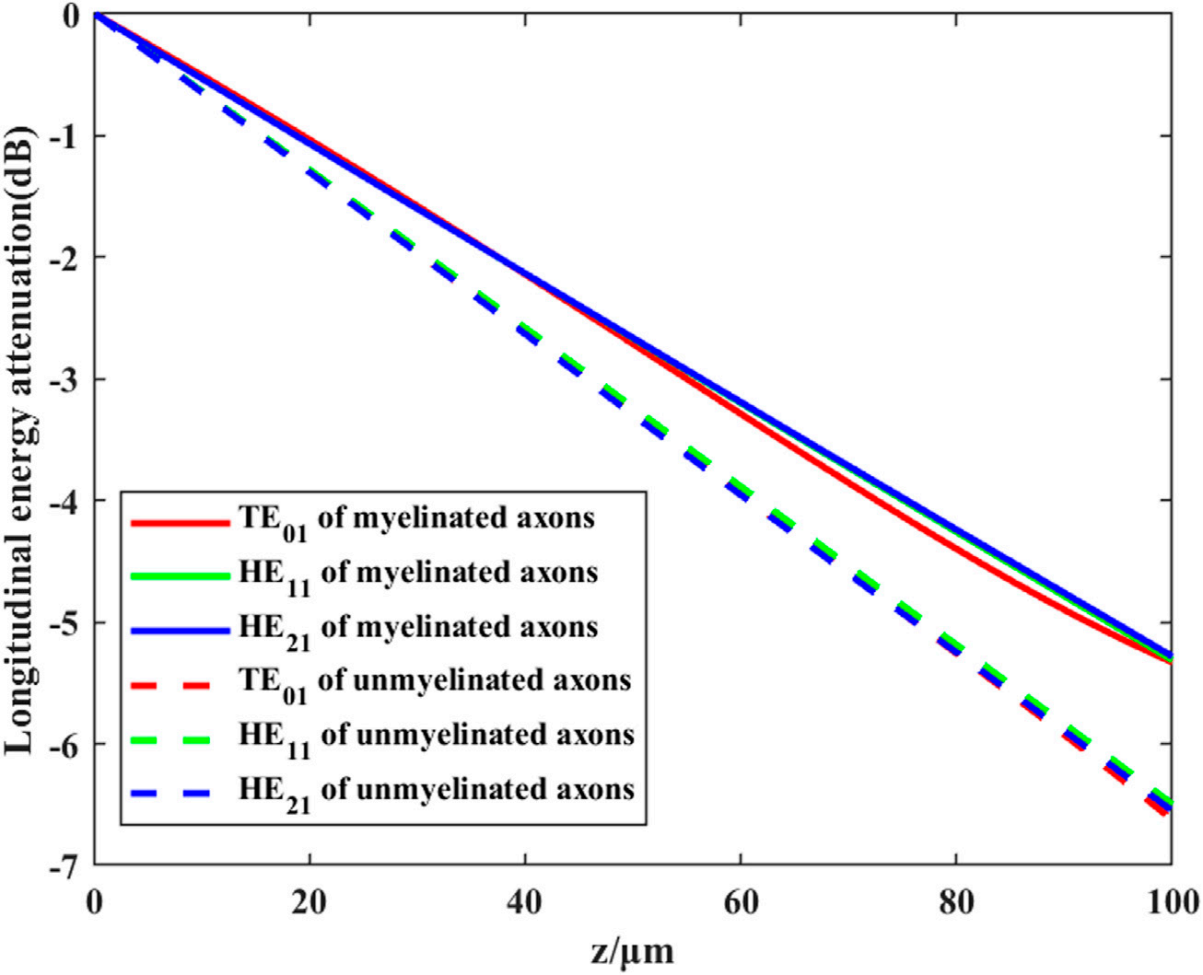
Transmission efficiency of different modes of THz-FIR waves in myelinated and unmyelinated nerve fibers at f = 30 THz.

The complex permittivity of a polar solution can be expressed as:

$$\varepsilon =\varepsilon^{\prime }-j\left(\varepsilon^{\prime \prime }+\frac{\sigma }{\omega }\right)$$ (19)

where $\varepsilon^{\prime \prime }$ is the imaginary part of the permittivity, representing the polarization loss in the nerve fiber. Meanwhile, σ is the electrical conductivity, representing the ohmic loss of the nerve fiber. During the simulation, we take $\varepsilon^{\prime \prime }=0.1$ , σ=30.9S/m . The normalized Poynting vector of the longitudinal transmission THz-FIR waves in the nerve fibers are shown in Figure 7. It can be seen that myelinated nerve fibers can greatly reduce the loss caused by the absorption of polar liquids, such as water.

**FIGURE 8:**
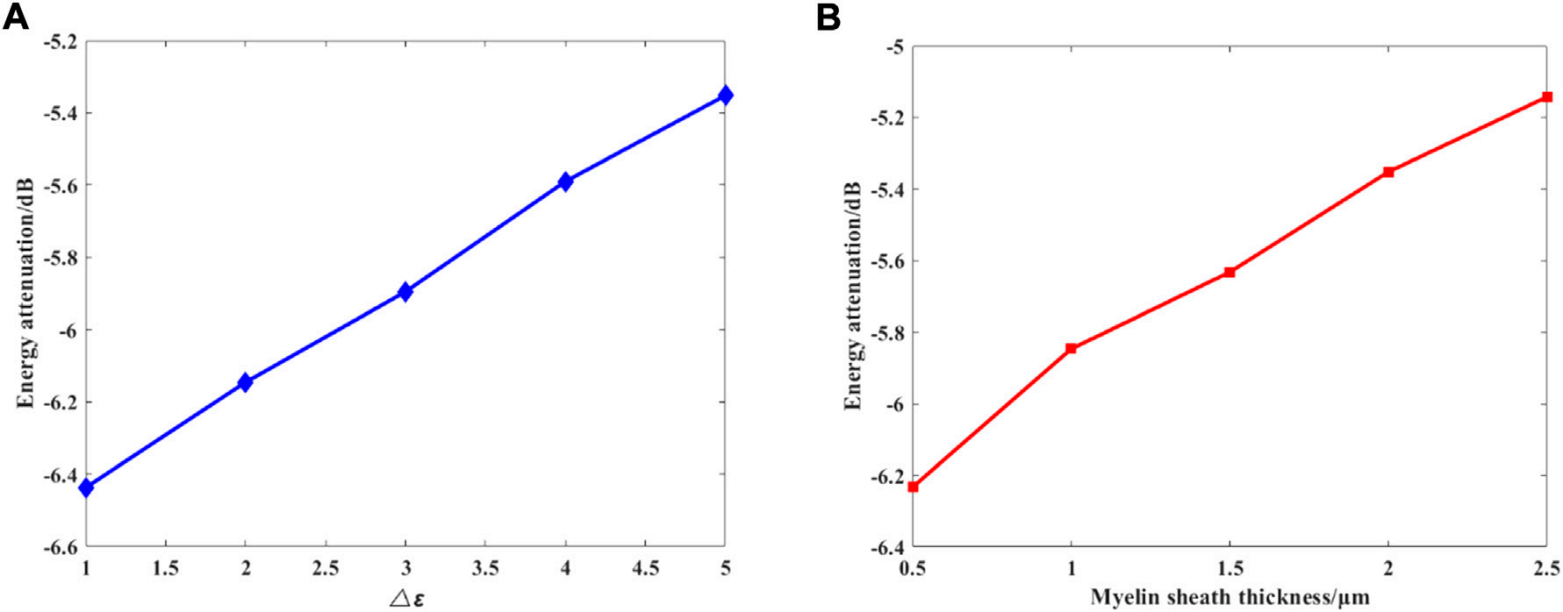
**(A)** Changes in the permittivity of the myelin sheath, and $d_{myelin}=2\mu m$ ; **(B)** changes in the thickness of myelin sheath, and Δε=5 .

We further investigate the effects of different myelin sheath thickness and dielectric constant on THz-FIR wave transmission. The theoretical calculation results of HE11 mode are shown in Figure 8.

Figure 8 shows the relationship between the maximum attenuation of THz-FIR waves traveling along the 100 μm long axon with the difference between the dielectric constants of the myelin sheath and the internal and external fluids, and the thickness of the myelin sheath when the frequency is 30 THz. It can be seen that with the increase of the permittivity and thickness of the myelin sheath, the attenuation of THz-FIR waves is greatly reduced. This indicates that the bearing capacity of the nerve fibers in different positions in the organism for the transmission of the THz-FIR wave is different. Thicker nerve fibers (e.g., squid’s spinal nerve) facilitate long-distance transmission of THz-FIR wave.

## Transmission characteristics at nodes of Ranvier

**FIGURE 9:**
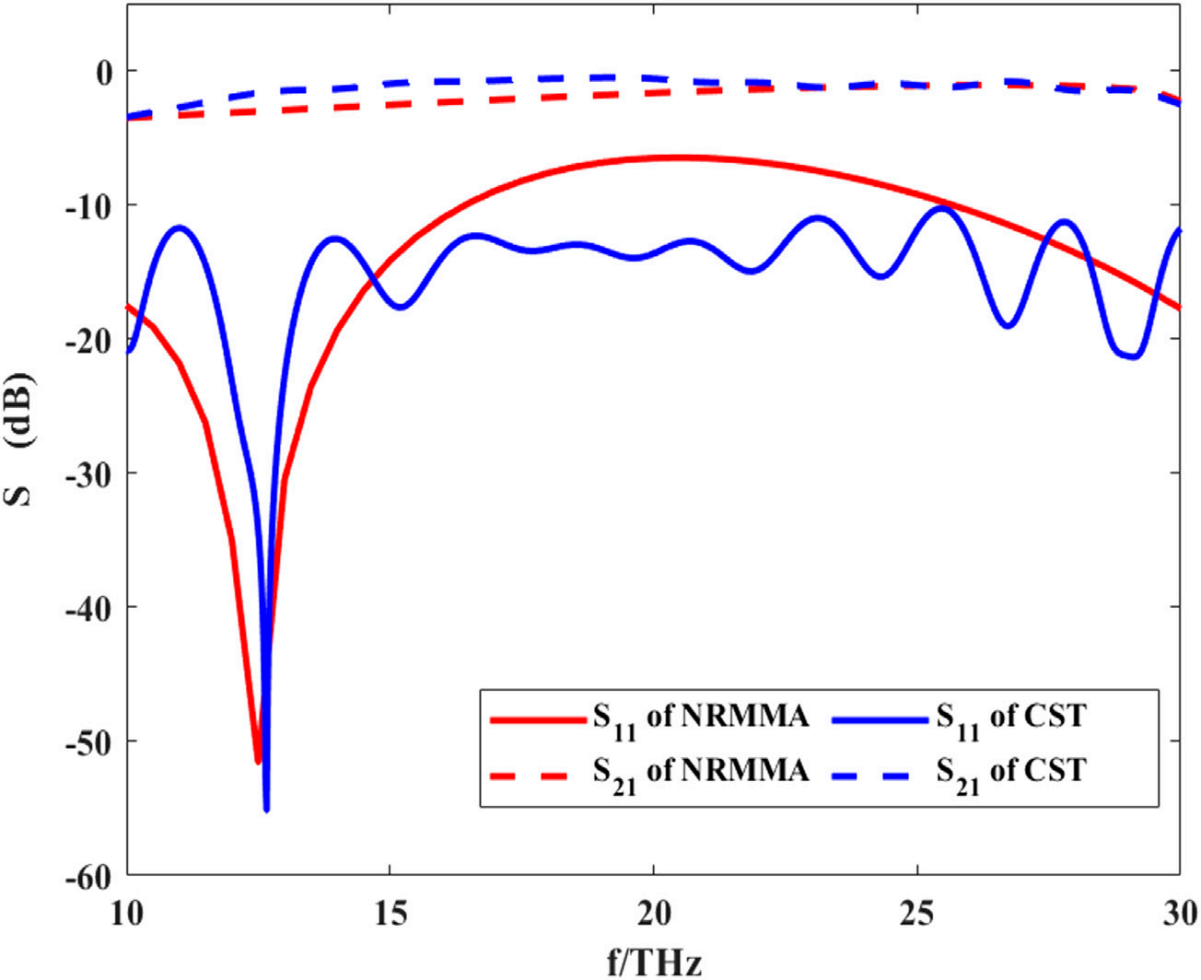
S-parameters of THz-FIR wave propagation at the nodes of Ranvier calculated by RNMMA and CST.

As an important physical parameter, the SM can be used to calculate the energy transfer process at the nodes of Ranvier. The calculation process of the SM is shown in Eqs 16 and 17. Here, we set the gap width of the nodes of Ranvier to 2 μm, the thickness of myelin sheath to 2 μm, the length of the axon to 100 μm, and the permittivity of three regions to $\varepsilon_{1}=4$ , $\varepsilon_{2}=9$ , and $\varepsilon_{3}=4.41$ , respectively. We then calculated the S11 curve and S21 curve of the node of Ranvier and compared them with the calculation results of the CST simulation software. The results are shown in Figure 9.

Compared with the result calculated by CST software, the results of the theoretical calculation are consistent with those from CST simulation software, which verifies the accuracy of our method. At f = 12.5 THz, S11 has the minimum value. This indicates that the reflection of THz-FIR transmission is the smallest at this time, which is mainly related to the width of the nodes of Ranvier. At the same time, S21 is greater than −5dB, which indicates that the THz-FIR wave has good transmission performance through the Ranvier junction. Meanwhile, the results also show that the S21 curve maintains a relatively stable value in the range of 10–30 THz and its value is greater than −5dB. This indicates that the node of Ranvier gap width (2 μm) has a weak influence on the transmission of THz waves. This happens because when the periodic gap width is much smaller than the wavelength of the THz-FIR wave, the THz-FIR wave can pass through the structure with minimal loss. This method provides theoretical support for further research on the interaction between THz-FIR waves and ion motion at the nodes of Ranvier.

## Conclusion

In this article, we investigated the propagation characteristics of THz-FIR waves at the nerve fibers and the nodes of Ranvier by theoretical analysis. We aimed to study the new physical mechanism of high-frequency signal transmission in the nervous system. First, the exact expressions of the guiding mode and radiation mode in the nerve fiber have been obtained by theoretical analysis and the dispersion curves of the transmission of the THz-FIR wave in the nerve fiber have been obtained by applying proper boundary conditions. Myelinated nerve fibers can support more modes of transmission than unmyelinated bare axons, which greatly enhances the possibility that THz-FIR waves can travel through nerve fibers. It was found from the calculation results of field distributions that the myelinated nerve fibers could concentrate the field energy within the myelin region to a great extent, thus avoiding the strong absorption of THz-FIR waves by the surrounding polar liquids. Meanwhile, the thickness and permittivity of myelin sheath had a considerable influence on the propagation of THz-FIR wave along the nerve fiber. With the increase in the thickness and permittivity of the myelin sheath, the attenuation of the terahertz wave in longitudinal transmission gradually decreases.

We established a pattern-matching algorithm for the first time, named RNMMA, which was used to investigate the electromagnetic wave propagation and reflection characteristics of longitudinal discontinuities at the axon and the nodes of Ranvier. The accuracy of our algorithm has been verified by comparing it with the calculation results of CST.

## Data Availability

The original contributions presented in the study are included in the article/Supplementary Material; further inquiries can be directed to the corresponding authors.
